# Low tidal volume mechanical ventilation against no ventilation during cardiopulmonary bypass heart surgery (MECANO): study protocol for a randomized controlled trial

**DOI:** 10.1186/s13063-017-2321-9

**Published:** 2017-12-02

**Authors:** Lee S. Nguyen, Messaouda Merzoug, Philippe Estagnasie, Alain Brusset, Jean-Dominique Law Koune, Stephane Aubert, Thierry Waldmann, Jean-Michel Grinda, Hadrien Gibert, Pierre Squara

**Affiliations:** 1 Critical Care Medicine Department, CMC Ambroise Paré, 25-27 Boulevard Victor Hugo, 92200 Neuilly-sur-Seine, France; 2 Cardiac Surgery Department, CMC Ambroise Paré, Neuilly-sur-Seine, France; 3 Anesthesiology Department, CMC Ambroise Paré, Neuilly-sur-Seine, France

**Keywords:** Cardiopulmonary bypass, Postoperative pulmonary complications, Protective ventilation, Low tidal volume

## Abstract

**Background:**

Postoperative pulmonary complications are a leading cause of morbidity and mortality after cardiac surgery. There are no recommendations on mechanical ventilation associated with cardiopulmonary bypass (CPB) during surgery and anesthesiologists perform either no ventilation (noV) at all during CPB or maintain low tidal volume (LTV) ventilation. Indirect evidence points towards better pulmonary outcomes when LTV is performed but no large-scale prospective trial has yet been published in cardiac surgery.

**Design:**

The MECANO trial is a single-center, double-blind, randomized, controlled trial comparing two mechanical ventilation strategies, noV and LTV, during cardiac surgery with CPB. In total, 1500 patients are expected to be included, without any restrictions. They will be randomized between noV and LTV on a 1:1 ratio. The noV group will receive no ventilation during CPB. The LTV group will receive 5 breaths/minute with a tidal volume of 3 mL/kg and positive end-expiratory pressure of 5 cmH2O. The primary endpoint will be a composite of all-cause mortality, early respiratory failure defined as a ratio of partial pressure of oxygen/fraction of inspired oxygen <200 mmHg at 1 hour after arrival in the ICU, heavy oxygenation support (defined as a patient requiring either non-invasive ventilation, mechanical ventilation or high-flow oxygen) at 2 days after arrival in the ICU or ventilator-acquired pneumonia defined by the Center of Disease Control. Lung recruitment maneuvers will be performed in the noV and LTV groups at the end of surgery and at arrival in ICU with an insufflation at +30 cmH20 for 5 seconds. Secondary endpoints are those composing the primary endpoint with the addition of pneumothorax, CPB duration, quantity of postoperative bleeding, red blood cell transfusions, revision surgery requirements, length of stay in the ICU and in the hospital and total hospitalization costs. Patients will be followed until hospital discharge.

**Discussion:**

The MECANO trial is the first of its kind to compare in a double-blind design, a no-ventilation to a low-tidal volume strategy for mechanical ventilation during cardiac surgery with CPB, with a primary composite outcome including death, respiratory failure and postoperative pneumonia.

**Trial registration:**

ClinicalTrials.gov, NCT03098524. Registered on 27 February 2017.

**Electronic supplementary material:**

The online version of this article (doi:10.1186/s13063-017-2321-9) contains supplementary material, which is available to authorized users.

## Background

Ventilator-acquired pneumonia (VAP) is a common postoperative complication and accounts for a large part of post-cardiac surgery morbidity and mortality. Incidence of VAP depends on numerous factors, including pulmonary collapse and atelectasis during cardiopulmonary bypass (CPB), lowering of bronchial arterial blood flow and systemic inflammation response syndrome during and after CPB [1–6].

To date, the impact of mechanical ventilation during CPB is unknown. On the one hand, CPB allows blood oxygenation during cardiac surgery, regardless of heartbeat and oscillations, allowing the surgeon to operate without disturbance [7]. On the other hand, postoperative pulmonary complications appear to be more frequent when no mechanical ventilation is maintained while under CPB [8].

A recent meta-analysis identified oxygenation improvement after the weaning from CPB when low tidal volume (LTV) ventilation was maintained or after lung recruitment maneuvers (LRM), as compared to when there was no ventilation (noV) [9]. Furthermore, maintaining mechanical ventilation may reduce the inflammation response and tissue damage [10, 11]. As the design of previous studies did not include hard clinical endpoints such as respiratory complications, death or length of stay, there is as yet no evidence for an unquestionable standardized strategy of lung protection during CPB and there are no scientific recommendations on whether mechanical ventilation has to be maintained during cardiac surgery or not, notably between LTV ventilation and noV [12]. Last, the nature of the intervention makes it hard for protocol investigators to blind the investigators to the intervention, explaining why all trials assessing mechanical ventilation are open-labeled [13].

The trial - low tidal mechanical ventilation against no ventilation during cardiopulmonary bypass heart surgery (“MECANO”) - aims to prove the superiority of the LTV compared to the noV strategy during CPB in cardiac surgery, to decrease postoperative respiratory complications, assessed by hard clinical endpoints using a double-blind design.

## Methods/design

### Methods

**Fig. 1:**
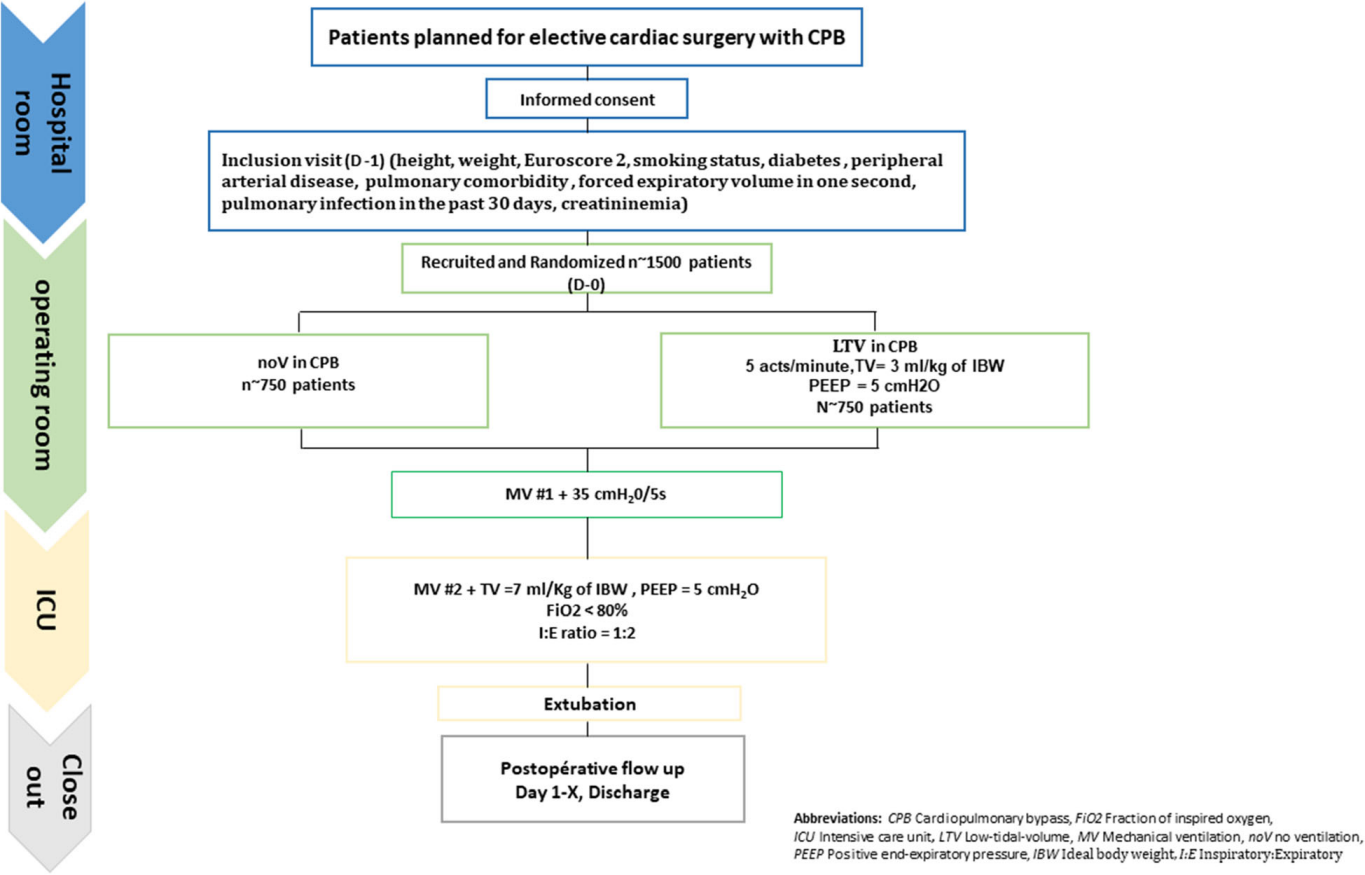
Study design. *CPB* cardiopulmonary bypass; *FiO2* fraction of inspired oxygen; *ICU* intensive care unit; *LTV* low-tidal volume; *TV* tidal volume; *MV* mechanical ventilation; *noV* no ventilation; *PEEP* positive end-expiratory pressure; *IBW* ideal body weight; *I:E* Inspiratory:Expiratory

The MECANO trial is a single-center, double-blind, non-pharmacological, randomized, controlled trial comparing two mechanical ventilation strategies, LTV and noV, during cardiac surgery with CPB (Fig. 1).

### Population

All patients aged more than 18 years and planned for elective cardiac surgery with CPB are eligible. Thoracoscopic surgery procedures are excluded. The inclusion and exclusion criteria are shown in Table 1.

**Table 1:** Eligibility criteria

Inclusion criteria	Exclusion
Signed informed consent	Unplanned surgery (emergency)
Age >18 years	Pregnant women
Planned surgery with CPB	Inability to understand the informed consent
Affiliation to French Social Security	Impossibility to wean CPB at the end of surgery
	Thoracoscopic surgery

*CPB* cardiopulmonary bypass

#### Endpoints

The primary endpoint is a composite of overall death, early respiratory failure defined as partial pressure of oxygen (PaO2)/fraction of inspired oxygen (FiO2) ratio <200 at 1 hour after arrival in the intensive care unit (ICU), heavy oxygenation support (defined as a patient requiring either non-invasive ventilation, mechanical ventilation, or high-flow oxygen) at 2 days after arrival in the ICU or VAP as defined by the Center of Disease Control [14]. Secondary endpoints are those composing the primary endpoint with the addition of pneumothorax, CPB duration, volume of postoperative bleeding, red blood cell transfusions, requirements for revision surgery, length of stay in the ICU and in the hospital and total hospitalization costs. Patients are followed until hospital discharge (Table 2).

**Table 2:** Endpoints

	Endpoint
Primary	All-cause mortality
	Early respiratory failure (PaO2/FiO2 ratio <200 in the first hour after transfer to ICU after surgery)
	Late respiratory failure (heavy oxygenation support (non-invasive ventilation, high-flow oxygen or mechanical ventilation) 2 days after surgery)
	Ventilator-acquired pneumonia
	Early pneumonia (early or ventilator-acquired)
Secondary	All-cause in-hospital mortality
	Early respiratory failure
	Heavy oxygenation support
	Pneumonia
	Length of stay in the ICU (days) after the initial cardiac surgery)
	Length of stay in the hospital (days) after the initial cardiac surgery
	Cost of hospitalization (euros)
Other	Revision surgery (requirement for any revision cardiac surgery after the initial surgery)
	Pneumothorax (diagnosed on chest x-ray or CT-scan occurring after the initial surgery)
	Postoperative bleeding (mL)
	Cardiopulmonary bypass duration (minutes)
	Red blood cells transfusion (units)

*PaO2/FiO2* partial pressure of oxygen/fraction of inspired oxygen; *CT-scan* computerized tomography scanner

#### Randomization

Randomization is performed by the anesthesiologist in charge upon the patient’s arrival in the operating room using an external interactive web response system (IWRS). A 1:1 treatment ratio with blocks of various even size (to prohibit prior guessing of the allocation group) is used. Thus, physicians outside the operating room (i.e. study investigators) are not aware of the treatment arm. Any deviation from the protocol is recorded, as is the reason for deviation.

#### Intervention

The noV group receives no ventilation during CPB. The LTV group receives 5 breaths/minute with a tidal volume of 3 mL/kg and positive end-expiratory pressure (PEEP) of 5 cmH2O. Lung recruitment maneuvers are performed in both groups at the end of surgery when thorax is closed and on arrival in the ICU, with insufflation at +30 cmH20 for 5 seconds. In the ICU, the ventilation strategy is lung-protective: tidal volume = 6 mL/kg of ideal body weight, PEEP = 5 cmH2O, FiO2 set to obtain PaO2 between 200 and 250 mmHg, inspiration/expiration time ratio = 1:2. Other therapeutic approaches are left to the decision of the ICU intensivists.

#### Data collection

All data are recorded on a dedicated online case report form (CRF). Preoperative data are collected prior to surgery (age, height, weight, EuroSCORE 2, smoking status, diabetes mellitus, peripheral arterial disease, pulmonary comorbidity, forced expiratory volume in one second, pulmonary infection in the past 30 days and creatininemia). Variables linked to the surgery are type of procedure, duration of CPB (in minutes), number of red blood cell transfusions and numbers of and reasons for manual insufflation. Data collected on daily visits are systematically recorded for 3 days, including temperature, PaO2, FiO2, ventilation mode, hemoglobinemia, leucocytemia and quantity of bleeding. Endpoints described earlier and time to event are tracked throughout hospitalization with follow up maintained until hospital discharge (Table 3).

**Table 3:** Flow-chart: enrollment, interventions and evaluations

		Study period						
Time points^a^								
	Enrollment	Allocation/intervention		Post intervention			Follow up	Close out
	Preoprative visit	Before anesthesia	During surgery	Day 1	Day 2	Day 3	Day X	Hospital discharge
	(D-1 or D-2 before surgery)							
Enrollment								
Eligibility screen	X							
Informed consent	X							
Physical examination^b^	X							
History of previous disease^c^	X							
FEV (1) and FVC	X							
Euroscore 2	X							
Creatininemia (μmol/L)	X							
Randomization		x						
Intervention								
Type of procedure			x					
CPB (in minutes)			x					
Number of red blood cell transfusions			x					
Number of and reasons for manual insufflation			x					
Assessments								
Temperature				x	x	x		
PaO2				x	x	x		
FiO2				x	x	x		
Ventilation mode				x	x	x		
Hemoglobinemia				x	x	x		
Leucocytemia				x	x	x		
Quantity of bleeding				x	x	x		
Collection of data on the occurrence of primary^d^ and secondary^e^ endpoints				x	x	x	x	x
Serious adverse events				x	x	x	x	x

*Abbreviations*: *CPB* cardiopulmonary bypass; *D* day; *FiO2* fraction of inspired oxygen; *PaO2* arterial oxygen tension; *FEV (1)* forced expiratory volume 1; *FVC* forced vital capacity

^a^Time points: enrollment, interventions and assessments

^**b**^Physical examination: weight, height

^**c**^History of previous disease: diabetes mellitus, peripheral arterial disease, pulmonary comorbidity, pulmonary infection in the past 30 days

^d^Primary endpoints: overall death, early respiratory failure defined as PaO2/FiO2 ratio <200 at 1 hour after arrival in the ICU, heavy oxygenation support (defined as a patient requiring either non-invasive ventilation, mechanical ventilation or high-flow oxygen) at 2 days after arrival in the ICU or ventilator-acquired pneumonia as defined by the Center of Disease Control

^e^Secondary endpoints: pneumothorax, CPB duration, volume of postoperative bleeding, red blood cell transfusions, requirements for revision surgery, length of stay in the ICU and in the hospital and total hospitalization costs

#### Statistical considerations

Sample-size calculation is based on two-sided alpha error of 0.05 and 80% power. Based on respiratory insufficiency incidence after cardiac surgery, we anticipate that at least 25% of patients will present with postoperative respiratory complications. We expect a relative improvement in the incidence of the primary outcome of 20% between the two arms (odds ratio 0.8 in favor of the LTV arm as compared to the noV arm). The required sample size is then 720 patients per group, 1440 patients in total. Accounting for the attrition ratio, 1500 patients will be included. Interim analyses will be performed. The sample size will be recalculated after every analysis based on the conditional probability of the final outcome.

#### Data analysis

Patients will be analyzed following the intention-to-treat principle. Binomial regression eventually supplemented by modified logistic regression (Diaz-Quijano, BMC Medical Research Methodology 2012, 12:14) and survival regression will be performed for statistical analysis. Relative risks and hazard ratio with 95% confidence intervals and differences between medians with 95% confidence intervals will be calculated by bootstrapping (3000 iterations) when appropriate. Two-sided significance tests will be used throughout. We will infer a subgroup effect if the interaction term of treatment and subgroup is statistically significant at *P* <0.05.

#### Ethical approval and clinical trial authorization

The trial is conducted in adherence to the current version of the Helsinki Declaration, the French Law on Protection of Personal Information and the National Health Law. The Regional Ethics Committee has approved the study protocol, which was also approved by the French Data Protection Agency. The trial protocol is registered at ClinicalTrials.gov (NCT03098524). Patients are enrolled only after written informed consent has been obtained.

## Discussion

Postoperative pulmonary complications (PPCs) are common and serious complications after cardiac surgery [15], despite continuing improvements in CPB techniques and postoperative intensive care. They are broadly defined as conditions affecting the respiratory tract that can significantly impact on patient outcomes and health economics [16, 17].

During CBP, the lungs are under perfused, non-ventilated or supplied with low continuous ventilation, depending on the center protocol [18]. Clinical trials have suggested that preventive lung-protective ventilation may improve outcomes in patients undergoing high-surgery [16].

The goal of the MECANO trial is to compare the effects of no ventilation during CBP and LTV ventilation of 3 mL/kg with a PEEP of 5 cmH2O during CPB in cardiac surgery. We believe that the present study has several strengths. First, the number of patients to be included (n = 1500) is ambitious. Several previous studies aimed to prove the beneficial effect of protective ventilation in cardiac surgery [16]. However, most trials were insufficiently powered or biased, leading to high heterogeneity and lack of conclusive results [13, 19–31]. The CPBVENT trial (NCT02090205) aims to answer a similar question. Although multicenter by design, it is a single-blind study and focuses on indirect outcomes (PaO2/FiO2 ratio only) instead of harder clinical endpoints such as hospital-acquired pneumonia or death. This explains why the number of patients to be included in CPBVENT is smaller (n = 720 vs. n = 1500 in our study). Moreover, CPBVENT compares three ventilation strategies (no ventilation, continuous positive airway pressure (CPAP) and LTV ventilation), which may decrease the power of the study [13]. Finally, the inclusion criteria are stricter, decreasing the possibility to generalize the results.

Second, the MECANO trial addresses observer bias by using a double-blind design, with investigators only involved in the post-surgery setting. As such, they are never aware of the allocation arm of the included patients. Randomization ensures equity and balance between the two treatment strategies. In-hospital follow up allows for complete follow up of all patients and is sufficiently pertinent, as postoperative pulmonary complications are expected to happen within the hospital stay after surgery. Third, data on all variables that may account for the increased risk of postoperative pulmonary complications are collected at baseline, ensuring equivalence between the two treatment strategies.

Limitations of the MECANO trial include its single-center design, although the number of physicians accounts for a wide scope of practices, all in line with current guidelines. Second, the surgeon has the final say in the type of mechanical ventilation, i.e. he can stop any type of ventilation strategy during CPB as he sees fit. This bias is addressed by systematically collecting the reason for and number of times that this may happen. Analyses will be performed on an intention-to-treat and per-protocol basis. Moreover, this will generally show how feasible or not, a maintained LTV ventilation might be and guide clinical practice accordingly.

In conclusion, the MECANO trial should help determine whether low-tidal ventilation is superior to no-ventilation, during cardiac surgery with CPB. It is the first double-blind trial of this kind, with a large population and focusing on hard clinical endpoints (Table 2, Additional file 1).

## Trial status

The first patients were randomized on 1 May 2017. The inclusion of participants is ongoing and is expected to continue until 15 April 2019.

## Additional file

Additional file 1: SPIRIT 2013 Checklist: Recommended items to address in a clinical trial protocol and related documents. (DOCX 47 kb)

## Abbreviations

CPAP: Continuous positive airway pressure
CPB: Cardiopulmonary bypass
CRF: Case report form
FiO2: Fraction of inspired oxygen
ICU: Intensive care unit
LRM: Lung recruitment maneuvers
LTV: Low-tidal volume
noV: No ventilation
PaO2: Partial pressure of oxygen
PEEP: Positive end-expiratory pressure
PPCs: Post-operative pulmonary complications
VAP: Ventilation-acquired pneumonia
